# Stochastic microbial dispersal drives local extinction and global diversity

**DOI:** 10.1098/rsos.231301

**Published:** 2024-05-15

**Authors:** Miguel Garrido Zornoza, Namiko Mitarai, Jan O. Haerter

**Affiliations:** ^1^ The Niels Bohr Institute, University of Copenhagen, 2100 Copenhagen, Denmark; ^2^ Constructor University, Bremen, Germany; ^3^ Leibniz Centre for Tropical Marine Research, Bremen, Germany; ^4^ Department of Physics and Astronomy, University of Potsdam, Potsdam, Germany

**Keywords:** coexistence, dispersal, trade-off, stochastic, competition

## Abstract

Airborne dispersal of microorganisms is a ubiquitous migration mechanism, allowing otherwise independent microbial habitats to interact via biomass exchange. Here, we study the ecological implications of such advective transport using a simple spatial model for bacteria–phage interactions: the population dynamics at each habitat are described by classical Lotka–Volterra equations; however, species populations are taken as integer, that is, a discrete, positive extinction threshold exists. Spatially, species can spread from habitat to habitat by stochastic airborne dispersal. In any given habitat, the spatial biomass exchange causes incessant population density oscillations, which, as a consequence, occasionally drive species to extinction. The balance between local extinction events and dispersal-induced migration allows species to persist globally, even though diversity would be depleted by competitive exclusion, locally. The disruptive effect of biomass dispersal thus acts to increase microbial diversity, allowing system-scale coexistence of multiple species that would not coexist locally.

## 1. Introduction

Microbes are involved in global nutrient and energy cycles and constitute a key functional group in the ocean’s food web [1–3]. For example, half of the oxygen in the atmosphere is generated by photosynthetic bacteria [2]. There are a total of ∼ 10^30^ prokaryotes on Earth [4], of which ∼ 10^29^ are oceanic bacteria [4] permanently hunted down by bacteriophages (short: ‘phages’, i.e. viruses that infect bacteria), which constitute their most common ‘predator’, or parasite [5–7]. Indeed, studies point to the ubiquity of viral infections [5,8], for example, 20–30% of marine bacteria are believed to be infected at any given time by phages [5]. Beyond regulating their host’s population and community structure [9–12], and despite their lack of metabolism, viruses also influence energy and nutrient cycles by modifying the microbial metabolism [7,13–16] and by directly impacting microbial mortality [15,17–23].

However important, the interplay between phages and bacteria, reflected in the size and complexity of their ecological network [12,24–26], is still poorly characterized. Mathematical modelling is a strong tool to unveil possible mechanisms that maintain microbial diversity. When considering large-scale aquatic ecosystems (e.g. [11,12]), much work is based on well-mixed models, where the competitive exclusion principle [27] dominates the coexistence rules. However, when the habitat is spatially structured, these rules are altered and a higher degree of diversity is allowed [28]. Metapopulation studies of predator–prey and host–parasite systems [29,30] have shown that migration between habitats can support global coexistence by reintroducing locally extinct species from another habitat, but also trigger species extinction by provoking large-amplitude predator–prey oscillations.

In phage–bacteria systems, dispersal due to aerosol transport has the potential to cover vast distances [31,32], before returning to the surface via wet or dry deposition [33]. Indeed, models suggest ∼ 10^24^ particles containing bacteria to be emitted globally every year into the atmosphere [34] with residence times estimated to vary from days to weeks [34]. In this sense, we can consider the atmosphere as a vector that promotes microbial dispersal across otherwise spatially disconnected habitats [35], with the potential ability to impact an ecosystem’s composition [28,35–37] despite the much lower advected concentration numbers as compared to surface populations [33,34,38,39].

In this work, the focus is on the atmosphere’s role in biomass transport and its potential to shape microbial community structure, in particular, the predator–prey system composed of phages and bacteria. We view the atmosphere as a habitat where these microbes are carried around stochastically as sessile organisms and can only survive transiently, that is, do not replicate but suffer from decay. Passive dispersal thus provides a migration mechanism for these microorganisms, which are transported across the surface, considered to be physically homogeneous and spatially subdivided. Our goal is to understand the ecological implications of such a system. For this, we here develop a simple two-layer neutral dispersal [40] model. Within the framework of our model, we first address the baseline dynamics emergent from these dispersal-mediated stochastic biomass fluxes, which effectively connect surface habitats. Extinction within a given habitat as a result of stochastic migrations is shown to be of utmost importance in shaping community structure. Second, we study the implications of such dynamics on competition and diversity, focusing, for simplicity, on a two-phage system sharing a common bacterial host. We find biodiversity to self-organize, even under conditions where competitive exclusion would rule out coexistence.

## 2. Methods

### 2.1. Model concept

**Figure 1.**
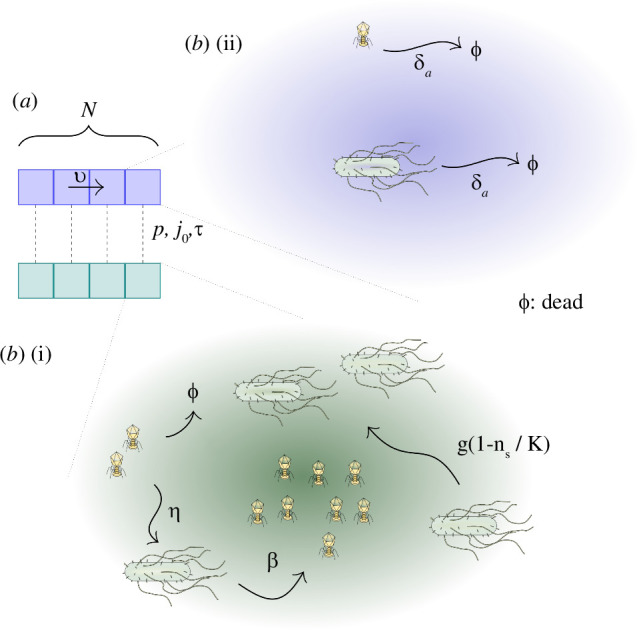
Schematic representation of the spatial model. (*a*) Two different types of biomass transport are modelled: continuous advective flow, v , in the upper layer and vertical stochastic transport, described with three parameters $\left(p,j_{0},\tau \right)$ , that allows particles to ‘jump’ across layers, effectively coupling them. (*b*) Bacteria undergo a layer-dependent palette of events. (i) When in the surface, they are exposed to phage predation ( η ), and have access to enough nutrients to grow logistically ( g,K ). (ii) As an aerosol, they avoid predation but are exposed to a much higher decay rate ( $\delta_{a}$ ), accounting for the more extreme conditions found in the atmosphere. Aerosolized phages follow an analogous behaviour, whereas when on the surface, they predate and multiply ( η,β ), as well as decay ( $\delta_{s}$ ), requiring the presence of the host to survive.

Our quasi-one-dimensional model consists of two coupled one-dimensional layers, or linear habitats (see figure 1*a*), each subdivided into N sites. In the lower layer, each of these sites constitutes a surface habitat, where basic chemical or physical nutrients are sufficiently available and species can replicate and interact. These discrete surface habitats are connected only by airborne dispersal via the upper layer. This layer, representing the atmosphere, is only relevant for directed advective transport, as well as decay, disregarding replication or predation processes. Microbes thus only spread passively. The exchange between the two layers is enabled through vertical stochastic population fluxes.

Our model is, therefore, a hybrid between a continuous formulation, taking place for replication and decay in the surface layer as well as transport within the atmospheric layer, and stochastic processes, which occur when biomass is transported vertically. Decay is possible in both layers and in practice likely more pronounced in the atmospheric layer due to UV radiation exposure there [41].

### 2.2. Model formulation

In both linear habitats, the spatial coordinate x is discretized into N positions $x_{i}=i\Delta x$ , with the integer i∈[0,N) and the spatial extent of each habitat Δx .

#### 2.2.1. Surface dynamics

Within each grid box at a given discrete position $x_{i}$ , the lower-layer (surface) bacterial and phage population densities, $n_{s}(x_{i},t)$ and $m_{s}(x_{i},t)$ , respectively, are assumed to follow the set of Lotka–Volterra equations [42]


(2.1) $$\begin{array}{rl} \frac{dn_{s}}{dt} & =gn_{s}\left(1-\frac{n_{s}}{K}\right)-\eta \;n_{s}m_{s}\;, \end{array}$$

(2.2) $$\begin{array}{rl} \frac{dm_{s}}{dt} & =(\beta -1)\eta n_{s}m_{s}-\delta_{s}m_{s}\;, \end{array}$$

where we have dropped the explicit reference to spatial and temporal coordinates for simplified notation. In equations (2.1) and (2.2), η is the reaction kernel or the adsorption rate, and captures the reaction-limited nature of phage infection, that is, how often viruses can both find and infect their host; β is the phage replication number, typically referred to as burst size; $\delta_{s}$ the phage decay rate (see figure 1*b*(i) for a zoom into these local dynamics). Furthermore, in this predatory dynamics, we tacitly assume lytic [43] phages and well-mixed populations within each grid box. We thus ignore the high degree of spatial heterogeneity one could find in different environments [44–47] and its associated ecological impact [48,49]. We put the focus on bulk and large-scale behaviour, setting our scale of interest to a few metres.

The zeroth trophic level, representing basic chemical or physical energy sources, is not explicitly modelled. Instead, in equation (2.1), we assume bacteria follow logistic growth [50] with maximum growth rate g and a constant maximum carrying capacity, K . K is thereby specific to the environmental context of the system. We do not have an explicit bacteria decay term here because, in a deterministic system with species described in terms of population densities, the bacterial decay rate can be absorbed into the growth rate without loss of generality.

#### 2.2.2. Dynamics in the upper layer

We assume the transport of bacteria and phages present in the upper layer to take place by passive advection following the atmospheric flow. The population densities will thus follow the advection-reaction equation, that is,


(2.3) $$\begin{array}{rl} \frac{\partial n_{a}}{\partial t} & =-v\frac{\partial n_{a}}{\partial x}-\delta_{a}^{n}n_{a}\;, \end{array}$$

(2.4) $$\begin{array}{rl} \frac{\partial m_{a}}{\partial t} & =-v\frac{\partial m_{a}}{\partial x}-\delta_{a}^{m}m_{a}\;. \end{array}$$

In contrast to the surface layer, here we consider explicit positive $\delta_{a}^{n}$ and $\delta_{a}^{m}$ , of similar magnitude, representing the respective bacterial and phage decay rates in the atmosphere (figure 1*b*(ii)). v is the horizontal advection velocity, which we have set constant for simplicity.

#### 2.2.3. Vertical transport

Vertical transport is taken as a stochastic process. We build our parameterization of microorganism emissions on literature [51] based on an empirical dust emission formulation [52,53]. The key concept we take from this work is a critical threshold value for near-surface wind speed above which vertical transport is finite. As a threshold phenomenon, this wind-driven emission mechanism is considered to be intermittent. Conceptualizing, aerosolization events are taken as discrete on-/off-like processes that occur with some activation probability, p , a parameter that aims to capture the frequency with which the wind speed is above the given threshold. Since this threshold might depend on the type of terrain, a given value of p qualitatively encompasses both the wind regime in a given location as well as the roughness of the surface over which the air is moving. In our model, we treat p as a free parameter.

On top, we shall consider net vertical exchanges to be qualitatively similar to eddy-like mixing, and triggered by this critical threshold on wind speed. In turbulent eddies, often parameterized as down-the-gradient fluxes [54], downward fluxes are fully correlated with emission events since mass transport is modelled to act in a similar way to molecular diffusion, but at a much larger scale. Consequently, in our scheme, the net exchange of biomass across layers is not only intermittent but, when finite, proportional to the vertical density gradient in each particular column. The proportionality constant, or rate of exchange, $j_{0}$ , is also taken as a free parameter.

The duration of these discrete events remains to be defined. In reality, their length is not necessarily fixed but, for simplicity, we here consider a constant exchange time scale, T . Consequently, when active, vertical transport will have a typical duration of τ∼T . This exchange is set to be independent among species, and of stochastic nature, that is, it will only happen with our probability, p . For simplicity, this is taken to be independent of the state of the system in the previous time interval.

#### 2.2.4. Vertical transport algorithm

Biomass exchange between the two levels of a specific column is therefore temporally discontinuous or intermittent and regulated by the three free parameters (see figure 1*a*):

1.
p , the probability of having a particle flux between layers for a given duration;
2.
τ , the duration of this intermittent biomass exchange between layers;
3.
$j_{0}$ , the rate at which these microbes are exchanged when vertical transport is active.

Summarizing, each location experiences biomass fluxes between same-column grid boxes with a frequency set by p . When this flux is active, particles are exchanged at a constant rate $j_{0}$ for a time τ . In practice, we evaluate the net intermittent bacterial and phage fluxes between layers, $j_{n}(x,t)$ and $j_{m}(x,t)$ , respectively, with the following algorithm:

For each horizontal position $x_{i}$ and each species separately, with i∈[0,N) , draw α∈U[0,1) , then, during the time t→t+τ

— If α≤p , the downward and upward fluxes are set to $j^{down}=j_{0}\cdot n_{a}(x_{i},t)$ and $j^{up}=j_{0}\cdot n_{s}(x_{i},t)$ , with the net flux being $j_{n}(x_{i},t)=j^{up}-j^{down}$ .
— If α>p , there is no biomass exchange in column $x_{i}$ , that is, $j_{n}(x_{i},t)=0$ .

This allows for particle fluxes along the gradient, leading to discrete aerosolization or colonization events whose frequency, duration and magnitude are free parameters. The final system reads as


(2.5) $$\begin{array}{rl} \frac{\partial n_{s}}{\partial t} & =gn_{s}\left(1-\frac{n_{s}}{K}\right)-\eta n_{s}m_{s}-j_{n}\;, \end{array}$$

(2.6) $$\begin{array}{rl} \frac{\partial m_{s}}{\partial t} & =(\beta -1)\eta n_{s}m_{s}-\delta_{s}m_{s}-j_{m}\;, \end{array}$$

(2.7) $$\begin{array}{rl} \frac{\partial n_{a}}{\partial t} & =-v\frac{\partial n_{a}}{\partial x}-\delta_{a}^{n}n_{a}+j_{n}\;, \end{array}$$

(2.8) $$\begin{array}{rl} \frac{\partial m_{a}}{\partial t} & =-v\frac{\partial m_{a}}{\partial x}-\delta_{a}^{m}m_{a}+j_{m}\;. \end{array}$$

Parameter values for equations (2.5)–(2.8) can be found in table 1 (appendix A). For the simulation of this model, population densities are randomly initialized across the spatial system (see appendix A), which is solved with periodic boundary conditions.

#### 2.2.5. Extinction threshold

Even though we work with population densities, we consider species populations to be integer numbers. For this, we manually introduce an extinction threshold equal to one individual per grid box, that is, $\rho_{ext}\equiv 1/V$ , V being the volume of the box. Whenever a particular trajectory drops below $\rho_{ext}$ , the species’ population is immediately set to zero.

#### 2.2.6. Main model assumptions

It is informative to briefly summarize the main model assumptions and limitations:

1. Net vertical fluxes are proportional to vertical population density differences. Furthermore, vertical transport is considered to be completely uncorrelated among species, that is, each species undergoes vertical transport independently of the other species. The model can be extended to study the effect of correlated emissions/depositions among species.
2. The frequency ( p ) and rate ( $j_{0}$ ) of aerosolization or deposition events are assumed to be equal among species. This could be generalized to allow for the empirically observed species-specific parameters [55–59].

## 3. Results

### 3.1. Core dynamics

We now look at the emerging dynamics of such a system and the resulting ecological consequences. However, the full complexity of the spatial model is better understood in terms of the behaviour of its individual components.

#### 3.1.1. Single column

Let us first focus on single surface grid-boxes in two different scenarios in order to decouple: (i) the effect of biomass loss to the upper layer (negative fluxes) and (ii) the effect of biomass gain from upstream sources into a populated habitat (positive fluxes).

##### 3.1.1.1. Negative fluxes

Let us consider habitats to be completely disconnected from their neighbours, that is, once aerosolized, microbes are advected and lost. Vertical transport thus represents a net loss of surface biomass. We shall study the effect of these intermittent fluxes by looking at the deviation of the population densities with respect to some deterministic expectation. More concretely, for this, let us look at the limit where fluxes are continuous ( τ→0 ) and a fraction p of the time vertical transport is active. This limit yields the deterministic equations


(3.1) $$\frac{dn_{s}(t)}{dt}=gn_{s}\left(1-\frac{n_{s}}{K}\right)-\eta n_{s}m_{s}-pj_{0}\cdot n_{s}\;,$$

(3.2) $$\frac{dm_{s}(t)}{dt}=(\beta -1)\eta n_{s}m_{s}-\delta_{s}m_{s}-pj_{0}\cdot m_{s}\;.$$

It is known that systems (equation 2.1)–(equation 2.2) and (equation 3.1)–(equation 3.2) contain a globally stable coexistence fixed point [60] (appendix B). Further, upon small perturbations, their transient relaxation to the steady state can be described as a stable spiral (appendix C), that is, population density trajectories oscillate back to this coexistence fixed point. This yields a clear picture of the deterministic dynamics when habitats are nudged away from their steady state. In this frame, the study of discrete transport comes from comparing the behaviour of system (equation (3.1))–(equation (3.2)) against its stochastic counterpart—equations (equation (2.5)) and (equation (2.6)) with $j^{down}=0$ —as we move between the well-mixed ( p→0 ) scenario, where surface habitats are isolated, and the continuous flux ( p→1 ) case. This is done by looking at the behaviour of both systems for different $\left(p,j_{0}\right)$ values while keeping the product $pj_{0}=\text{const}$ . Since the parameters p and $j_{0}$ appear as a product in (equation (3.1))–(equation (3.2)), they effectively behave as one, and any combination fulfilling this restriction is equivalent in the deterministic system, that is, it will result in the same dynamics. For convenience, let us now define an ‘equivalent deterministic line’ (EDL) as that where $pj_{0}=\text{const}$ . If we move along an EDL, as we tend to either p=1 or p=0 , both continuous and discrete scenarios converge, but, we will show that the behaviour is rather different for finite p owing to stochasticity in fluxes and the existence of the extinction threshold.

**Figure 2.**
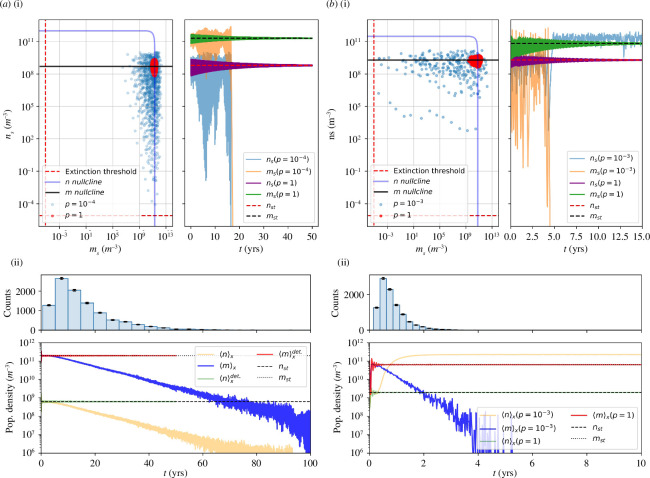
Negative fluxes. (*a*) (i) Right. Time series of phage and bacteria population densities for deterministic ( p=1 ) and stochastic ( $p=10^{-4}$ ) vertical transport cases along the same equivalent deterministic line, $p\cdot j_{0}\cdot \tau =1.6\times 10^{-5}$ . Here, the oscillations reach the bacterial extinction threshold, after which the phage population density decays to zero, as they need their host to survive. Left. Explored phase space of a C→E transition. (ii) Population density average of $10^{3}$ and $10^{4}$ independent surface habitats for the same deterministic ( p=1 ) and stochastic ( $p=10^{-4}$ ) transport cases, respectively. Decaying trajectories, corresponding to the stochastic case, are the result of individual extinction events, counted in the histogram above (shared time axis). (*b*) (i,ii) Analogously to the previous case, a C→F transition is shown ( $p=10^{-3}$ ) and compared to its deterministic limit ( p=1 ) in the $p\cdot j_{0}\cdot \tau =2\times 10^{-4}$ equivalent deterministic line. Notice the decay time-scale difference with respect to the previous case. Here, decay events happen much faster.

**Figure 3.**
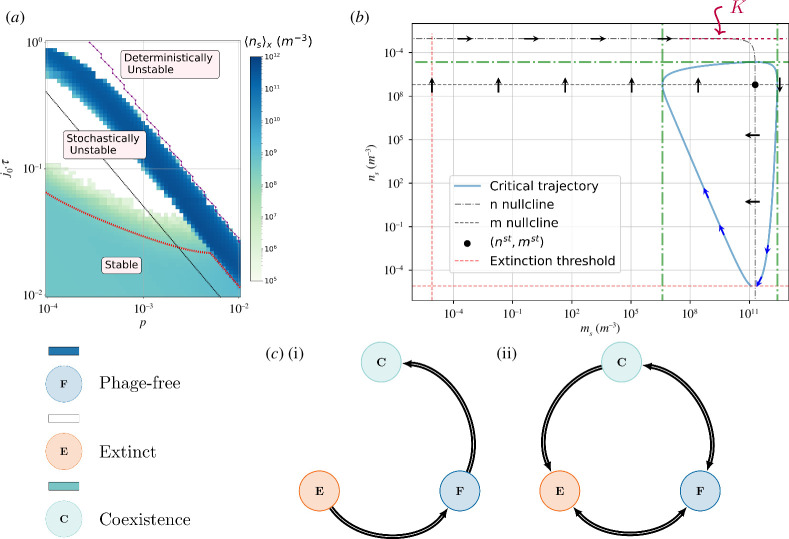
Single habitat dynamics. (*a*) Phase diagram of the single grid-box system. Each pixel represents the bacterial population average over $10^{3}$ independent habitats at t=150 years . Below, colours are matched to the corresponding state of the system: C , coexistence; F , phage-free; E , extinct. The black dashed line is an example equivalent deterministic line. (*b*) Positive flux framework imposed by the critical trajectory. Any migration event, or concatenation of migration events, must push the trajectory into the area encompassed by the critical trajectory, otherwise, the habitat is doomed to cross the extinction threshold. This limits, for example, the manner in which phages can migrate into a habitat populated by their host without driving that same habitat to extinction. (*c*) Available transitions of individual habitats. (i) Without an extinction threshold, a habitat can only undergo E→F bacteria-mediated transitions and F→C phage-mediated transitions. C is therefore an absorbing state. (ii) Diagram of new dynamical possibilities. These constitute the aggregate of negative and positive fluxes onto a system with a finite extinction threshold.

When subject to intermittent fluxes, stochastic effects appear: the original transient oscillatory relaxation to the coexistence fixed point is now substituted by trajectories which systematically show sustained oscillations and become unstable in some regions of the EDL. As seen in figure 2*a*,*b*(i) (right), the continuous case converges to the coexistence fixed point $\left(n^{st},m^{st}\right)$ from equations (B 3) and (B 4) whereas trajectories subject to intermittent biomass fluxes oscillate, eventually driving the system to extinction. We find two distinct types of extinction: (i) bacterial extinction, thus also causing parasite extinction; (ii) phage extinction and bacterial survival. Negative fluxes therefore open the possibility for coexistence among phages and bacteria ( C ), phage-free ( F ) and extinct ( E ) habitats, where neither species is present. In figure 2*a*,*b*(i) (left), we show the distinct explored phase space of each of the two types of extinction events. Given the stochastic nature of these fluxes, one particular realization might significantly differ from another. For this, the ensemble average of many independent repetitions is depicted in figure 2*a*,*b*(ii) (bottom). By counting the number of extinctions in time, figure 2*a*,*b*(ii) (top), we find the extinction rate to be exponentially distributed, and dependent on the pair ( $p,j_{0}$ ). A broader analysis is represented in the $\left(p,j_{0}\right)$ phase diagram of figure 3*a*, in which the region where these transitions take place is mapped out. We can define three distinctly different $\left(p,j_{0}\right)$ regions. (i) A deterministically unstable region, where even in a phage-free environment logistic growth cannot sustain bacterial biomass loss to the upper layer. The system is therefore driven to extinction. This is a deterministic prediction (appendix D). The connected purple dots in figure 3*a* show the limit where a finite density fixed point is still feasible. (ii) A stable region with permanent coexistence among phages and bacteria. Discontinuous fluxes make the system oscillate incessantly. (iii) A stochastically unstable region where, depending on the manner biomass is lost, that is, the ( $p,j_{0}$ ) pair, habitats with coexisting species transition either towards a phage-free state (dark blue diagonal) or an extinction of both phages and bacteria (white upper left, also in figure 3*a*). The border region between stable and extinct states shows a colour gradient reflecting the different extinction rates within the stochastically unstable region (a lower population average among independent habitats indicates the mixed presence of both extinct and populated habitats). This underlines the temporal aspect of the phase diagram, that is, for sufficiently long times, every habitat is susceptible to suffer from a concatenation of events that drive it to extinction. As seen in the diagram (and previously explored in figure 2*a*,*b*(ii) (top)), this time scale is set by $\left(p,j_{0}\right)$ .

##### 3.1.1.2. Positive fluxes

Next, we analyse the system’s response against the stochastic migration of phages or bacteria into a habitat populated by either bacteria or both phages and bacteria. From the system (equation 3.1)–(equation 3.2) nullclines we can see that, when pushed beyond some critical trajectory (figure 3*b*, in blue), the system will deterministically cross the extinction threshold. The critical trajectory thus provides a conceptual basis to understand the migration dynamics in our system. For example, if the bacterial habitat is in its carrying capacity, K , any migration attempt on the phage’s side will result in a complete deterministic depletion of the host (as seen from the phase portrait), thereby driving the full habitat to extinction. However, if the bacterial habitat has not yet reached the carrying capacity, it is possible for the parasite to successfully migrate, that is, push the trajectory into a region within the area encompassed by the critical trajectory. The transition F→C is thus conditional. Based on this discussion, we see that depending on the migrated population, a particular habitat can transition to any of the three possible states (except E→C ). This yields a more complex dynamical scenario as compared to the initial unique absorbing state (see figure 3*c*). Let us now look at the consequences of such a scenario in a connected system.

### 3.1.2. Multiple columns

#### 3.1.2.1. Connectivity effect

**Figure 4.**
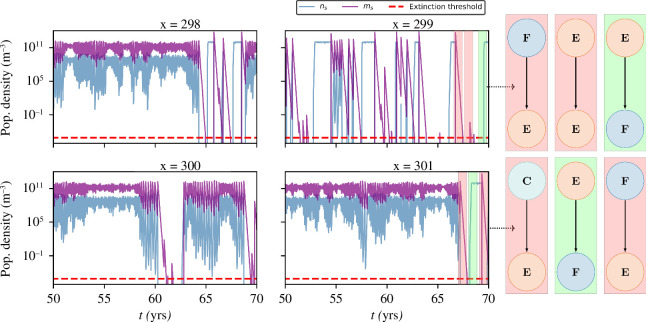
Connectivity effect. Time evolution of neighbouring surface habitats of a system with N=1000 and L=0.5 . Here, $p=10^{-4}$ and $j_{0}=6\times 10^{-3}\;s^{-1}$ . Different examples of extinction mechanisms are depicted to the right—for example, migration of the parasite, driving the system into the extinction threshold ( F→E , two cases shown); phages migrating into an empty habitat, thus causing them to decay ( E→E ); bacteria migrating into empty habitats and colonizing them ( E→F , two cases shown); an unstable habitat upon negative fluxes ( C→E ). For this particular spatial system, ∼93% of phage migrations into an *F* habitat resulted in extinction, reflecting the role of the critical trajectory introduced by the extinction threshold.

**Figure 5.**
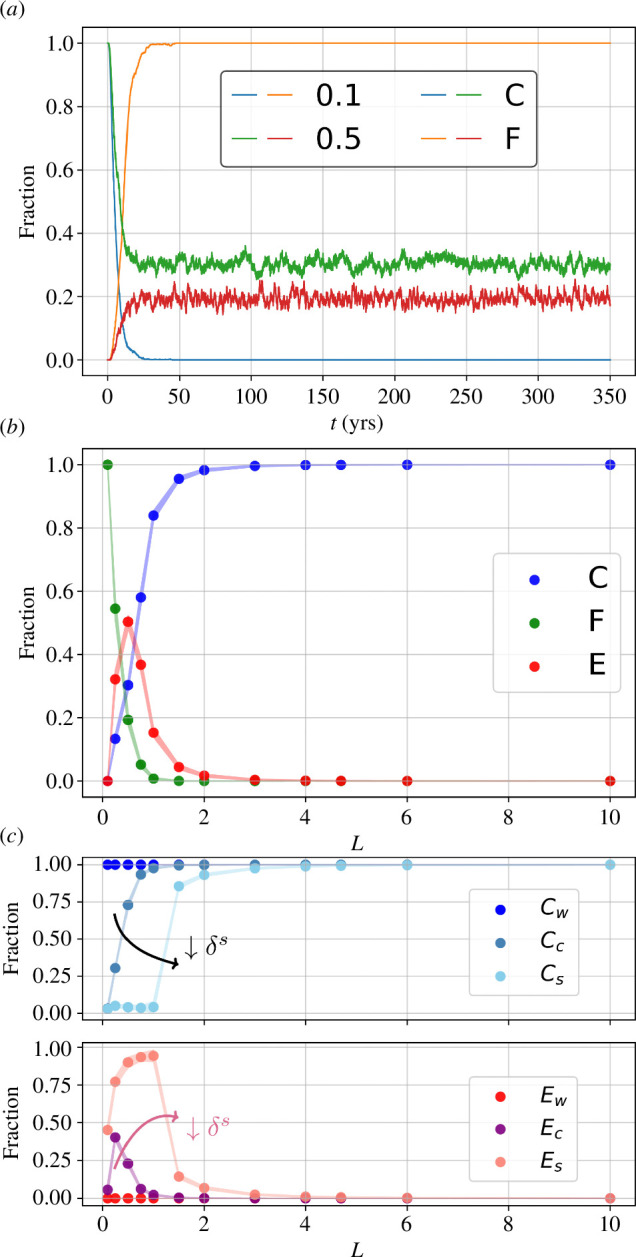
Steady states. (*a*) Relaxation to a steady state. Example trajectories for the L=0.1,0.5 cases for C and F fractions. Fluctuations correspond to transitions of individual habitats to a different state. (*b*) The fraction of columns in each state is a function of the system’s connectivity, L . Here, trajectories are simulated for 350 years with periodic boundaries until a steady state is ensured. From this, we neglect the first 50 years and compute the mean. Scattered points are complemented with lines of width equal to 2⋅σ , to exemplify the signal noise and thus the rate at which columns transition between states. (*c*) Steady-state dependence on the phage decay rate. Analogously to the previous case, we now show the steady-state C and E fractions in three spatial systems, each containing either the control (c), the weaker (w) or the stronger (s) phage.

We now focus on the $\left(p,j_{0}\right)$ region of the phase diagram where coexistence states become extinct upon negative fluxes in the single column case, that is, C→E transitions (white zone in the stochastically unstable region). Let us study their collective behaviour by allowing a finite degree of connectivity, that is, biomass emissions will get advected a finite fraction of the system length, L , before decaying, and thus dynamically ‘interact’ with downstream locations. This length is defined in a simple way, to provide a clear operational definition (see explicit derivation in appendix F). In short, it gives the distance, $x^{*}$ , an emitted flux of magnitude K would travel before its density reaches the extinction threshold, $\rho_{ext}$ , if p=0 . This is, the only biomass loss in the upper layer comes from the decay component, $\delta_{a}$ . This scenario yields the relation


(3.3) $$x^{*}=L\cdot N\cdot \Delta x=-\frac{v}{\delta_{a}}\cdot \ln\left(\frac{\rho_{ext}}{K}\right)\;,$$

with N being the total system size. This length is effectively controlled with $\delta_{a}$ , which is chosen as a tuning parameter to modulate the system’s connectivity. In figure 4, we show individual contiguous habitats of a connected system with L=0.5 . From this, we see that, when biomass is allowed to disperse, the spatial system simultaneously splits into the three possible states, thus surviving local extinctions. Locally, each habitat is susceptible to transition from one state to another while the global system self-organizes into a statistical steady state. Two examples are shown in figure 5*a*. Even though first neighbours are uncorrelated, the fraction of the spatial system belonging to either state is a function of the system’s connectivity ( L ), figure 5*b*, reflecting the effect of dispersal distance for coexistence.

### 3.2. Implications for competition and diversity

The new dynamical possibilities (figure 3*c*(ii)) drive the spatial system towards a new set of steady states (figure 5*a*,*b*). Fundamentally, these configurations might not only depend on the biogeographic connectivity, L , but also on the system’s response to biomass fluxes, that is, the way trajectories converge back to the coexistence fixed point. Since this response is set by the deterministic parameters $\left(g,\beta ,\eta ,\delta_{s},K\right)$ , these steady states might be sensitive to a change in, at least, one of them. Interestingly, these parameters are also a measure of fitness, or competitive ability. A higher competitive trait for the phage, such as a bigger burst size, β , or a lower decay rate in the surface layer, $\delta_{s}$ , might even be detrimental, since, by changing the system’s convergence to the steady state, it could increase the chance of crossing the extinction threshold, and thus alter the habitat’s longevity. This line of thought underlines the non-trivial effects intra-population variability might have on the spatially structured habitat, and the complexity of understanding the net role of intrinsic or system-specific parameters. We now look at the dynamical role intrinsic parameters have in the spatial steady states, and the implications for competition and diversity. For the latest, we focus on the simplest extension of our study, that is, we introduce an extra phage which infects the same host and thus represents a direct competitor.

#### 3.2.1. Dynamical role of deterministic traits

Let us focus, for simplicity, on the aforementioned decay rate, $\delta_{s}$ . To understand the grounds of the conceptualized competition–longevity trade-off, we briefly go back to the system (equation 2.1)–(equation 2.2) and summarize the effect of $\delta_{s}$ in an isolated deterministic system.

First, from a linear stability analysis of system (equation 2.1)–(equation 2.2) (appendix C), we can show that the decay time scale of small perturbations, $\tau_{per}$ , is proportional to $\delta_{s}^{-1}$ . Systems with fitter viruses (smaller $\delta_{s}$ ) will thus take longer to fall back into their steady-state population densities. This might allow future fluxes to further amplify an initial departure from the steady state.

Second, given the existence of an extinction threshold, the stability of coexistence states is also related to the amplitude of their oscillations. This happens to increase for lower values of $\delta_{s}$ (see appendix G). Consequently, the stability of the habitat decreases for systems with stronger (smaller $\delta_{s}$ ) viruses.

#### 3.2.2. Effect of phage decay rate in the spatial system

Having the effect of $\delta_{s}$ on local population dynamics in mind, let us now look at the behaviour of the connected system in the two distinct scenarios of increasing and reducing by 10-fold the phage decay rate in the surface, $\delta_{s}$ . A comparison between these two independent spatial systems and the one with untouched $\delta_{s}$ value (control) is depicted in figure 5*c*. We can see that, for any given value of the system’s connectivity, L , the number of E habitats increases (decreases) for the stronger (weaker) phage case. A better competitive ability, having a clear local destabilizing effect, has detrimental effects on a spatial level. Intrinsic parameters thus clearly modify the spatial steady state and might therefore have an impact on global competition and diversity.

#### 3.2.3. Competition of two phage types in a spatial system

Let us now evaluate direct competition among phages with distinct competitive abilities. Examining the effect of phage migration into an isolated C habitat populated by the competitor we see that, as expected, competitive exclusion applies and the stronger phage takes over (appendix E). In the following, we demonstrate that the full spatial model can allow the global coexistence of these competing phage species.

**Figure 6.**
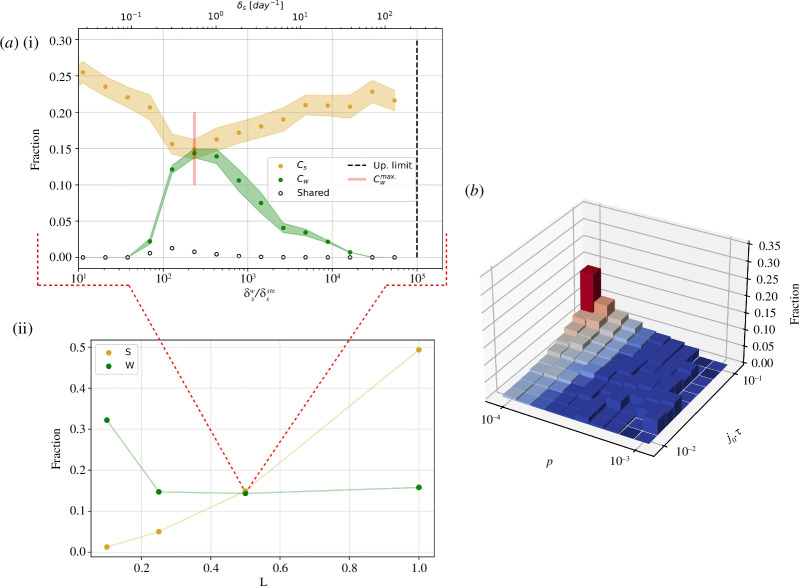
Direct competition of two phage types. (*a*) (i) For a fixed pair $(p,j_{0})=(0.1,3.2)\times 10^{-3}$ , we compute the fraction of habitats occupied by the weaker phage strain in the steady state , $C_{w}$ , for $\delta_{s}^{w}/\delta_{s}^{str.}\in [10^{1}\text{–}10^{5}]$ . From this, we take $C_{w}^{max.}=\max(C_{w})$ . The black dashed line marks the upper deterministic limit for the feasibility of the coexistence fixed point. (ii) Analogous analysis for different system connectivities, L . A clear transition appears as a function of L , from a dominance of the stronger phage to a dominance of the weaker competitor. The low fraction of habitats shared by both phages (white scattered dots) points towards local competitive dynamics acting on a much faster time scale than habitat connectivity. (*b*) Effective fitness landscape. We systematically estimate the optimal $\delta_{s}$ value for the weaker phage strain for different $\left(p,j_{0}\right)$ pairs for L=0.1 . This can be considered a measure of the effective fitness, as opposed to the intrinsic fitness, measured only from the deterministic parameters. Red shades indicate a dominance of the weaker strain, that is, a higher number of habitats occupied by it than its stronger competitor.

To study direct competition, we choose an arbitrarily small non-zero value of $\delta_{s}$ (the absolute zero would be biologically unfeasible) for the stronger phage, $\delta_{s}^{str.}$ . We then simulate the spatial system for a range of $\delta_{s}$ values for the weaker phage, $\delta_{s}^{w}$ , with $\delta_{s}^{w}\geq \delta_{s}^{str.}$ . This is initially done for a fixed $\left(p,j_{0}\right)$ pair and different connectivities, L . In figure 6*a*(i), we show the ability of the weaker phage to coexist in the spatial system. This coexistence is only possible in a given range of $\delta_{s}^{w}$ values, establishing a limit to how similar the weaker competitor can be in order for coexistence to be achieved. We also find the existence of an optimal decay rate value that maximizes the fraction of occupied sites by the weaker competitor, to the detriment of the stronger. That is, even though competitive exclusion applies and competitive dynamics act on a much faster time scale than biomass transport (see figure 6*a*(i) white dots), the weaker phage indirectly affects the number of habitats where the stronger competitor is present, thus creating new competitive dynamics. Furthermore, we learn that not only does the intrinsically less fit strain manage to coexist in the spatial system, but, for low connectivities, it even dominates over its stronger competitor (figure 6*a*(ii)). Fitness is therefore not fully determined by the intrinsic deterministic parameters, but also by the biomass transport regime, and thus the aggregate context of the particular habitat.

In figure 6*b*, we calculate the weaker phage species’ optimal decay rate for a given $\left(p,j_{0}\right)$ region to illustrate this idea. This same exercise can be done for the rest of the system’s intrinsic parameters, such as the phage’s burst size, β , or the bacterial growth rate, g , in order to understand the role each parameter plays on a global scale.

## 4. Discussion

In our simple two-layer model, the predatory bacteria–phage system we study is subject to discrete, intermittent, wind-driven gain and loss of biomass corresponding to migrations from upwind habitats and local aerosolization events, respectively. The stochastic fluxes introduced by such gain and loss processes provoke sustained oscillations in the population densities, observed in otherwise stable systems when subject to demographic noise [61]. These oscillations push individual habitats far from their coexistence fixed point, occasionally driving them to extinction. On a spatial level, the system reaches a new balance between local extinctions and dispersal-mediated migration, leaving a finite fraction of habitats either unpopulated or phage free. Overall, dispersal comes in as a source of instability as well as a driver for global microbial persistence in locally ephemeral habitats. Furthermore, these baseline dynamics are revealed to be crucial for microbial diversity. Even though competitive exclusion applies within individual habitats, conditions exist where, by persisting longer in local habitats, weaker phage strains manage to coexist in the spatial system and even dominate to the detriment of the stronger competitor, that is, indirectly reducing its presence in habitats where the first are not present.

In the framework of the model, inter-specific differentiation in competitive ability and its consequent change in habitat longevity is suggested to be important in allowing multi-species coexistence. This differentiation, however, seems to only be allowed if bounded, qualitatively aligning with the limiting similarity suggested by Tilman [28]. There are, however, a few elements linked to the particularities of the phage–bacteria system of study. (i) Whereas the limiting similarity concept suggests the existence of an upper bound to the fitness distance for species coexistence, we here observe that not only adjacent competitors (in our case the two phage species) are not allowed to be too close, but also too far from each other. We thus find that coexistence is allowed within a fitness interval, that is, there is also a lower bound to the fitness distance. (ii) Within this interval, there is an optimal fitness value, where the number of inhabited habitats by the weaker competitor is maximized. This is, however, not the highest possible value the competitor could have in order to coexist. This introduces the interesting idea of not having a clear evolutionary strategy for the weaker competitor. (iii) The spatial presence of the stronger phage decreases with the presence of the weaker counterpart, even though competitive exclusion applies and competitive dynamics work at a faster time scale than migration. In consequence, competition not only takes place locally, but also via the re-arrangement of the spatial structure.

In the atmosphere, the fate of a microorganism is related to the aerosolization, atmospheric processing and deposition circumstances, such as the drying conditions upon aerosolization or deposition [62], atmospheric temperature and humidity [63–65], salinity (osmostic pressure) [66,67], UV exposure [41] and nutrient availability [35]. All these traits likely represent environmental dispersal filters, a role supported by the suggested non-neutrality of dispersal [40,68–70], affecting the travel distance and survival rates, or the biogeographic connectivity. On top, we note that, despite comparably harsh conditions, the atmosphere has been proposed as a habitat where microorganisms can be metabolically active and grow [71–74] as well as contribute to physical [75–77] and chemical [78,79] transformations, potentially modifying cloud formation processes [76,77,80] and thereby affecting the hydrological cycle [81] and Earth’s global energy budget. These are all mechanisms susceptible to affect system-specific parameters such as the typical dispersal distance or the growth rate in a species-dependent manner. Our case study thus constitutes a proof of concept of the role microbial dispersal can play for community longevity and diversity.

## Figures and Tables

**Figure 7.**
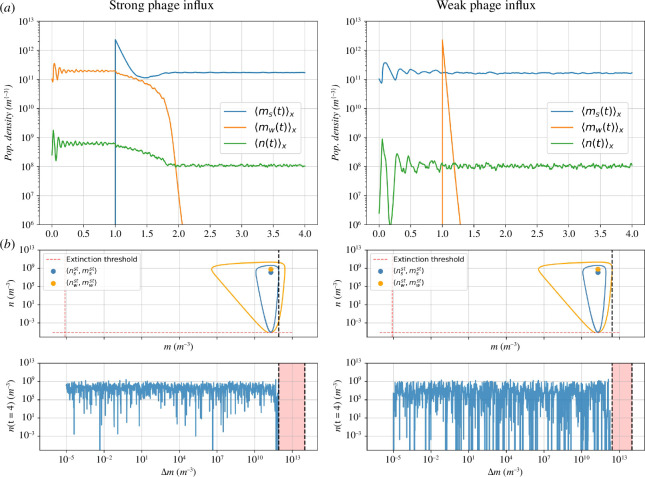
Two phages experiment. (*a*) Ensemble average of independent single habitats. At t=1 year , a different migrated phage quantity, Δm , arrives at each habitat—in all cases, the stronger phage takes over. (*b*) Bacterial population densities at t=4 years as a function of the migrated population, Δm . Each point is an independent habitat. On top, we draw the respective critical trajectories and fixed points for weaker and stronger phages.

**Figure 8.**
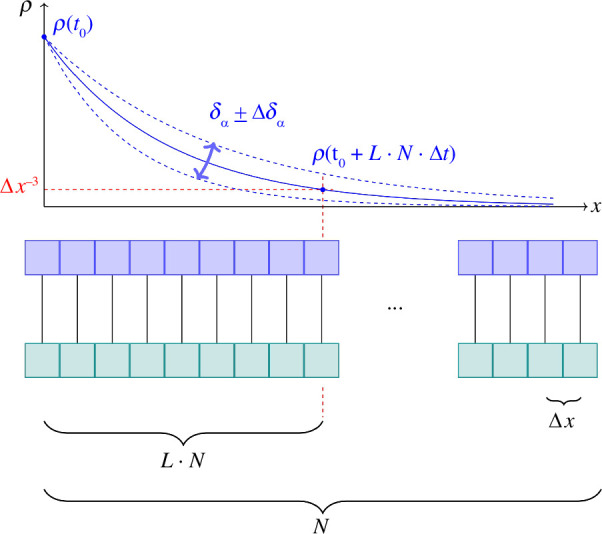
Signal length. Diagram of the system parameters relevant to define a given signal length, L , and the effect of the decay rate, $\delta_{a}$ .

**Figure 9.**
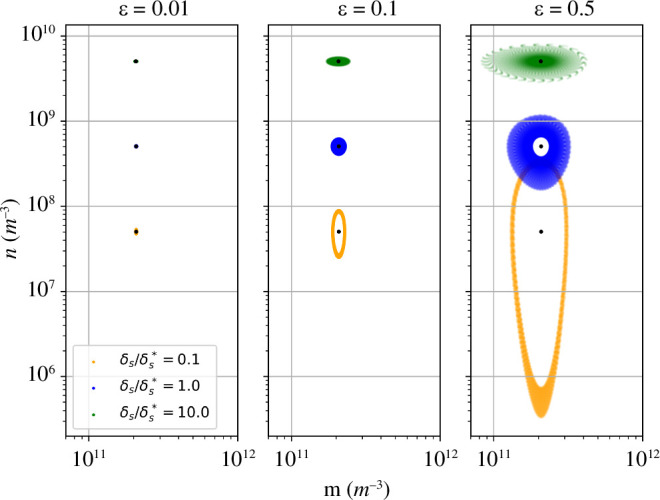
The effect of competitive traits. Response to different biomass influx of three independent single-habitat phage–bacteria systems with distinct parasite intrinsic fitness. This is here measured from the phage’s decay rate in the surface, $\delta_{s}$ , which takes the values [0.1,1,10] (expressed in units of the experimental value $\delta_{s}^{*}$ [85]). In each system, either species density, x , is initialized as $x(0)=(1+\epsilon )\cdot x^{st}$ for ϵ=0.01,0.1,0.5 (panels from left to right, respectively) and integrated for the same time.

## Data Availability

The full spatial model can be found at https://github.com/Mgarrizor/ecology_paper, and has been archived in the Zenodo repository [82].

## Appendix A. Simulation details

### A.1. Transport scheme

For our choice of spatial length scale, Δx=50 m , and typical diffusion coefficients for phages and bacteria (see caption in table 1), relevant timescales for crossing one habitat boundary are $∼\Delta x^{2}/D=10^{8}\text{–}10^{9}$ years and thus molecular diffusion can safely be neglected as a dominant transport mechanism. The choice of grid box size implicitly constrains the typical length scale of the phenomenon driving the vertical transport of biomass. We assume vertical transport events among neighbouring columns to be uncorrelated. In order for this assumption to hold the effective length over which a single mixing event takes place should not be bigger, or much smaller, than Δx . Turbulent eddies can vary greatly in size, from millimetres to hundreds of metres, suggesting that, in a more realistic setting, these compartments should be size distributed and their size should change in time. In this work, for simplicity, we assumed they are all of the same size.

**Table 1.** The diffusion coefficients used to roughly estimate the travelling time across grid-boxes are $\begin{array}{l} D_{n}\equiv \sqrt{D_{||}^{2}+D_{⊥}^{2}}=0.17\times 10^{-12}\;m^{2}\;s^{-1} \end{array}$ [83] for bacteria (*Escherichia coli*) and $D_{m}=2.76\times 10^{-12}\;m^{2}\;s^{-1}$ [84] for phages. The decay parameter in the upper layer, $\delta_{a}∼0.01\;{min}^{-1}$ , is shared among bacteria [63] (*E. coli*) and viruses [33, §2.4]. These parameters have not been picked as an attempt to fully characterize a particular system but to set the typical scales (the order of magnitude of the different rates). For this, we also used $\delta_{s}∼0.005\;h^{-1}$ [85] (T5-*E. coli* or order of magnitude from table), β∼100 [85] (order of magnitude from table) *E. coli*, $\eta ∼100\times 10^{-15}\;m^{3}\;h^{-1}$ [85] (order of magnitude from table) *E. coli*. For the growth rate and the carrying capacity, we assume the system to be embedded in an ocean-like context in terms of nutrient availability; with this in mind, we set $K∼10^{6}\;ml^{-1}=10^{12}\;m^{-3}$ and $g∼0.5\;d^{-1}$ [86–89].

system-specific parameters					
*K* (m^−3^)	*β*	*g* (d^−1^)	*η* (m^3^ d)	δ*s* (d^−1^)	δ*a* (d^−1^)
10^12^ [86–89]	100 [85]	0.5 [86–89]	2.14 × 10^−12^ [85]	0.12 [85]	864 [33,63]
spatial parameters					
∆*x* (m)	∆*t* (s)	*N*_*x*_	*N*_*y*_	*v* (m s^−1^)	*τ* (s)
50	50	1000	2	1	∆*t*

### A.2. Time step

In practice, we set τ to the numerical time-step when integrating (equation 2.5)–(equation 2.8). This is, τ=Δt . With this, in the algorithm, for every time step, we allow for vertical exchange at each column with probability p . This choice reduces the dimensionality of the explored parameter space, since τ is kept fixed throughout the study.

### A.3. Numerical scheme

Advection was in principle treated with a Lax–Wendroff scheme and a flux limiter correction to avoid spurious oscillations. However, in order to deal with ‘delta-like’ peaks from stochastic sources, which created density differences of up to $∼10^{12-14}$ in contiguous grid boxes, we decided to instead set the Courant number ( ≡v⋅Δt/Δx ) to unity, a trade-off that allowed us to better advect particles but constrained the time-step, and thus the numerical efficiency. For the time-stepping scheme, we used a fourth-order Runge–Kutta algorithm.

### A.4. Initial density profile

The initial density profile of species X is selected by drawing uniformly distributed values from the interval $\left[0,X^{st}\right)$ , where $X^{st}$ is the steady state calculated in equations (B 3) and (B 4).

## Appendix B. Lyapunov stable

We here show that the averaged equations (equation 3.1)–(equation 3.2) contain a globally asymptotically stable coexistence fixed point. For clarity, we write population densities in units of the carrying capacity, K , that is, $x\equiv n\cdot K^{-1}$ and $y\equiv m\cdot K^{-1}$ . By re-scaling the parameters accordingly, the equation reads as

(B 1)
$$\dot{x}=\tilde{g}x\cdot (1-x)-\tilde{\eta }xy-\tilde{c}x\;,$$

(B 2)
$$\dot{y}=(\tilde{\beta }-1)\cdot \tilde{\eta }xy-\tilde{\delta }y-\tilde{c}y\;.$$

The coexistence fixed points are

(B 3)
$$x^{st}=\frac{\tilde{\delta }+\tilde{c}}{\tilde{\beta }\tilde{\eta }}\;,$$

(B.4)
$$\tilde{\eta }y^{st}+\tilde{g}x^{st}=\tilde{g}-\tilde{c}\;.$$

A Lyapunov function, V(x,y) , exists for int $R_{+}^{2}$ . Commonly used trials have the form [60]

(B 5)
$$V(x,y)=H(x^{st},y^{st})-H(x,y)\;,$$

with

(B.6)
$$H(x,y)=x^{st}log(x)-x+y^{st}log(y)-y\;.$$

With this choice equation (B 5) is definite positive and $V({\vec{x}}^{st})=0$ . By making the slight modification

(B.7)
$$H(x,y)=x^{st}\log(x)-x+\frac{1}{\tilde{\beta }-1}\left(y^{st}\log(y)-y\right)\;,$$

we can see that $\dot{V}({\vec{x}}^{st})<0\;\forall \;\vec{x}\in R_{+}^{2}-\{{\vec{x}}^{st}\}$ . Given that

(B.8)
$$\frac{\partial V}{\partial x}=1-\frac{x^{st}}{x}\;,$$

(B 9)
$$\frac{\partial V}{\partial y}=\frac{1}{\tilde{\beta }-1}\cdot \left(1-\frac{y^{st}}{y}\right)\;,$$

we have

(B 10)
$$\dot{V}=(x-x^{st})\cdot (\tilde{g}(1-x)-\tilde{\eta }y-\tilde{c})\;+(y-y^{st})\cdot ((\tilde{\beta }-1)\cdot \tilde{\eta }x-\tilde{\eta }-\tilde{c})\cdot \frac{1}{\tilde{\beta }-1}\;=-\tilde{g}\cdot (x-x^{st}{)}^{2}\;.$$

In the last equality, we used (equation B 3) and (equation B 4). The coexistence fixed point is therefore globally asymptotically stable.

## Appendix C. LSA of the well-mixed system

System (equation 2.1)–(equation 2.2), which we shall label as ‘well-mixed’, is known to have a coexistence fixed point:

(C 1)
$$\begin{array}{rl} n_{s}^{st} & =\frac{\delta_{s}}{\eta (\beta -1)}\;, \end{array}$$

(C 2)
$$\begin{array}{rl} m_{s}^{st} & =\frac{g}{\eta }\left(1-\frac{n^{st}}{K}\right)\;, \end{array}$$

which is globally stable (appendix B) when feasible, that is, $n^{st}<K$ .^1^ Upon a small perturbation, the transient relaxation to the fixed point can be described as a stable spiral with a decay time scale of ∼21.7 years and an oscillation period of ∼25 days . This can be seen from a linear stability analysis. The Jacobian is

(C 3)
$$\left(\begin{array}{cc} g(1-2n_{s}/K)-\eta m_{s} & -\eta n_{s}\\ (\beta -1)\eta m_{s} & 0\; \end{array}\right)\;,$$

from which we obtain the eigenvalues, $\lambda_{i}$ . Given the system parameters (see appendix A, table 1) the eigenvalues are complex:

(C 4)
$$\lambda_{i}=\gamma \pm i\omega \;.$$

Here $\gamma =-\frac{1}{2}g\alpha$ and $\omega =\frac{1}{2}\sqrt{4\eta \delta_{s}m_{s}^{st}}$ , with $\alpha =n_{s}^{st}/K$ . This classifies the fixed point as a stable spiral with an oscillation period of

(C 5)
$$T=\frac{2\pi }{\omega }=0.07\;years∼25\;days,$$

and a time scale for the decay of small perturbations of

(C 6)
$$\tau_{per}=\frac{1}{|\beta |}∼21.7\;years.$$

Equations (3.1)–(3.2) can be mapped to system (2.1)–(2.2) with an effective growth rate

(C 7)
$$g^{eff.}=g-pj_{0}\;\;,$$

carrying capacity

(C 8)
$$K^{eff.}=\frac{g}{(g-pj_{0})\cdot K}\;,$$

and phage’s decay rate

(C 9)
$$\delta_{s}^{eff.}=\delta_{s}+pj_{0}\;.$$

Consequently, this analysis also applies to system (3.1)–(3.2).

## Appendix D. Phage-free survival limit

In the absence of bacteriophages and any sort of spatial structure (and therefore any grid-scale transport scheme), the bacterial density will, in its logistic growth, asymptotically reach the carrying capacity, K . However, when allowed to vertically move across layers a new contribution behaving as a sink might keep the system from reaching a finite density fixed point. This is the first layer of complexity with respect to the 0 -dimensional well-mixed case, that is, two ‘vertically’ aligned grid points where only bacteria are present and vertical transport fluxes are continuous. Analogously to (equation 3.1)–(equation 3.2), the system reads as

(D 1)
$$\begin{array}{rl} \frac{dn_{a}(t)}{dt} & =-\delta_{a}n_{a}+pj_{0}(n_{s}-n_{a})\;, \end{array}$$

(D 2)
$$\begin{array}{rl} \frac{dn_{s}(t)}{dt} & =gn_{s}\left(1-\frac{n_{s}}{K}\right)-pj_{0}(n_{s}-n_{a})\;. \end{array}$$

The steady state is

(D.3)
$$n_{a}^{st}=\frac{g}{\delta_{a}}n_{s}^{st}\left(1-\frac{n_{s}^{st}}{K}\right)\;,$$

(D.4)
$$n_{s}^{st}=\left(1-\frac{\delta_{a}\cdot pj_{0}}{(\delta_{a}+pj_{0})\cdot g}\right)\cdot K\;.$$

This yields an extra limit to the feasibility of coexistence:

(D 5)
$$\frac{\delta_{a}\cdot pj_{0}}{\left(\delta_{a}+pj_{0}\right)}<g\;,$$

in this case, exclusively related to the capability of bacteria to survive on their own.

## Appendix E. Migration experiments

Migration into a downstream habitat: 2 phages case. Here, we study the sytem’s response to positive fluxes for the following cases: (i) the stronger phage migrates into a C habitat inhabited by the weaker phage; (ii) the weaker phage migrates into a C habitat inhabited by the stronger phage. As expected, competitive exclusion applies, that is, the stronger phage dominates on both scenarios, as depicted in figure 7*a*. However, the critical trajectory of the migrating phage determines a value over which the host’s population density is doomed to cross the extinction threshold, and thus the whole habitat becomes extinct, as seen in figure 7*b*. This introduces the possibility for the weaker phage to drive its competitor to extinction.

## Appendix F. Signal length

*Operational definition*: To study the effect of local connectivity without interfering with the vertical biomass scheme, one could fine-tune the $\delta_{a}$ parameter. Let us define the signal length, L , as the maximum distance travelled by a biomass emission of magnitude equal to the system’s carrying capacity, K , in the p=0 case. From the tendency equation

(F 1)
$$n(t)=Ke^{-\delta \alpha T}\;,$$

(F 2)
$$t^{*}=L\cdot N\cdot \Delta t=-\frac{1}{\delta_{a}}\cdot \ln\left(\frac{\rho_{ext}}{K}\right)\;.$$

From this, we obtain L . A schematic illustration is depicted in figure 8. This is the definition of signal length that we shall use in the main text (§3.1.2.1). It is of importance to have in mind that only same pair $\left(p,j_{0}\right)$ cases can be compared when studying the ecological effects of the system’s connectivity, L .

## Appendix G. Amplitude of oscillations

The amplitude of the transient oscillatory behaviour back to the steady state is a function of the system’s deterministic parameters. Here, we explore, in an illustrative manner, how it depends on the values of the phage’s decay rate in the surface layer, $\delta_{s}$ . In figure 9, we see that, for lower values of the decay rate, and thus higher competitive ability, the oscillations approach the bacterial population density extinction threshold. This is taken as a sign for the decrease in stability of habitats with stronger viruses figure 9.
